# Increased visceral tissue perfusion with heated, humidified carbon dioxide insufflation during open abdominal surgery in a rodent model

**DOI:** 10.1371/journal.pone.0195465

**Published:** 2018-04-04

**Authors:** Jonathan P. Robson, Pavlo Kokhanenko, Jean K. Marshall, Anthony R. Phillips, Jan van der Linden

**Affiliations:** 1 Fisher and Paykel Healthcare, Auckland, New Zealand; 2 School of Biological Sciences, University of Auckland, Auckland, New Zealand; 3 Department of Molecular Medicine and Surgery, Karolinska Institute, Stockholm, Sweden; University of Pisa, ITALY

## Abstract

Tissue perfusion during surgery is important in reducing surgical site infections and promoting healing. This study aimed to determine if insufflation of the open abdomen with heated, humidified (HH) carbon dioxide (CO_2_) increased visceral tissue perfusion and core body temperature during open abdominal surgery in a rodent model. Using two different rodent models of open abdominal surgery, visceral perfusion and core temperature were measured. Visceral perfusion was investigated using a repeated measures crossover experiment with rodents receiving the same sequence of two alternating treatments: exposure to ambient air (no insufflation) and insufflation with HH CO_2_. Core body temperature was measured using an independent experimental design with three treatment groups: ambient air, HH CO_2_ and cold, dry (CD) CO_2_. Visceral perfusion was measured by laser speckle contrast analysis (LASCA) and core body temperature was measured with a rectal thermometer. Insufflation with HH CO_2_ into a rodent open abdominal cavity significantly increased visceral tissue perfusion (2.4 perfusion units (PU)/min (95% CI 1.23–3.58); p<0.0001) compared with ambient air, which significantly reduced visceral blood flow (-5.20 PU/min (95% CI -6.83- -3.58); p<0.0001). Insufflation of HH CO_2_ into the open abdominal cavity significantly increased core body temperature (+1.15 ± 0.14°C) compared with open cavities exposed to ambient air (-0.65 ± 0.52°C; p = 0.037), or cavities insufflated with CD CO_2_ (-0.73 ± 0.33°C; p = 0.006). Abdominal visceral temperatures also increased with HH CO_2_ insufflation compared with ambient air or CD CO_2_, as shown by infrared thermography. This study reports for the first time the use of LASCA to measure visceral perfusion in open abdominal surgery and shows that insufflation of open abdominal cavities with HH CO_2_ significantly increases visceral tissue perfusion and core body temperature.

## Introduction

The ability to perfuse sufficient oxygenated blood into a surgical wound plays a pivotal role in postoperative tissue recovery. Two postoperative complications that can arise from poor tissue oxygenation (PtO_2_) during surgery are surgical site infections (SSI) and anastomotic leaks. Reduced blood flow and oxygenation have been shown to significantly increase the chance of acquiring a SSI [1], reduce healing [2], and ultimately prolong the recovery of the patient. SSI’s account for up to 20% of all hospital acquired infections and affect approximately 5% of surgical patients, with rates approaching 20% in colorectal surgery [3]. These nosocomial infections impact not only the recovery time of the patient but also significantly increase the economic burden on health care systems, with 2–3 fold increases in patient care costs reported [4]. In addition, postoperative anastomotic leaks following colorectal surgery are a serious, unwanted complication that can lead to severe sepsis and death. Anastomotic leaks occur in approximately 2% of patients undergoing colorectal surgery with mortality rates approaching 5% [5], although rates as high as 12% have been recorded in patients undergoing colorectal cancer surgery [6]. While advances in surgery have reduced the incidences of these postoperative complications the rates and costs on both the patient and healthcare system remain high.

The causes of postoperative SSI’s and anastomotic leaks are numerous and not restricted to poor PtO_2_, however reduced intraoperative tissue perfusion may be a critical common factor. Increased tissue perfusion during surgery has been shown to significantly reduce anastomotic leaks [7] and PtO_2_ levels have been used as a predictor for these events [8]. Reduced perfusion impairs wound healing through a reduction in PtO_2_ and delivery of nutrients, metabolites and immune cells, and as such, low PtO_2_ as a measure of perfusion is also a predictor of SSI [1, 9]. Current systemic approaches to increase intra-operative local oxygen delivery have had limited success in reducing the incidence of SSI. Administration of high fraction of inspired oxygen (FiO_2_) have shown significant but moderate decreases in SSI following colorectal surgery [10], however administration of >60% FiO_2_ may be associated with an increased risk of adverse events, including mortality [11]. Moreover, other approaches such as induction of mild hypercapnia (partial pressure of expired CO_2_ 50mmHg) and volumetric variation of supplemental intravenous crystalloid fluid replacement during colonic resection surgery did not significantly reduce SSI rates [12] [13]. Importantly, these methods rely on adequate perfusion to deliver the desired changes to the local surgical area, which can be difficult to achieve during an operation. A preliminary study, looking at tissue perfusion during surgery, reported a decrease in tissue perfusion and oxygen tension following the administration of anaesthetics [14]. Therefore, a locally targeted intervention that could increase local wound perfusion, and thus oxygenation, may be more clinically successful in surgical patients.

The application of HH CO_2_ insufflation and its benefits in temperature maintenance during abdominal laparoscopic surgery have been extensively studied [15], but its use in open abdominal surgery is relatively novel. Recently, it has been shown that locally insufflating the abdominal cavity with HH CO_2_ significantly increased local PtO_2_ in a rodent model of open abdominal surgery [16]. An increase in local PtO_2_ was observed in comparison with both exposure to ambient air, and to insufflation of cold, dry (CD) CO_2_, indicating that humidity and temperature, in addition to the intrinsic properties of CO_2_, are important in regulating oxygenation levels. The findings were consistent with previous studies that have shown a correlation between temperature and perfusion [17, 18], however the relationship between humidity and perfusion has not been well established. To better analyse the effect HH CO_2_ has on perfusion we utilized a non-invasive laser speckle contrast analysis (LASCA) camera to measure perfusion changes of the exposed viscera in real-time. LASCA has been shown to provide rapid intraoperative assessments of relative blood flow and offers a robust method of measuring perfusion with high resolution [19]. In contrast to more conventional Laser Doppler methodologies LASCA is fast, inexpensive and allows for sensitive detection of flow speeds over a wider area [20]. The aim of this study is to test the ability of LASCA to measure perfusion changes resulting from insufflation of an abdominal cavity with HH CO_2_. We hypothesized that visceral perfusion would increase when HH CO_2_ was insufflated into an open abdominal cavity, compared with exposure to ambient air. In addition, we hypothesized that insufflation with HH CO_2_ would increase core and abdominal cavity temperature, as has been shown in previous publications [15, 21, 22].

## Materials and methods

### Animals

This study was approved by the University of Auckland Animal Ethics Committee (approval number 001603). Male Sprague Dawley rats (age: 8–10 weeks; 300-350g) were obtained from the University of Auckland Vernon Jensen Unit. Animals were bred and maintained within appropriate light- and temperature-control (14/10hr light/dark cycle, 22–24°C), and sustained on the Harlan Teklad global diet (Fisher Scientific, USA). Following the experiments, the rats were euthanized via cervical dislocation while still anaesthetised.

### Anaesthesia and monitoring

**Fig 1:**
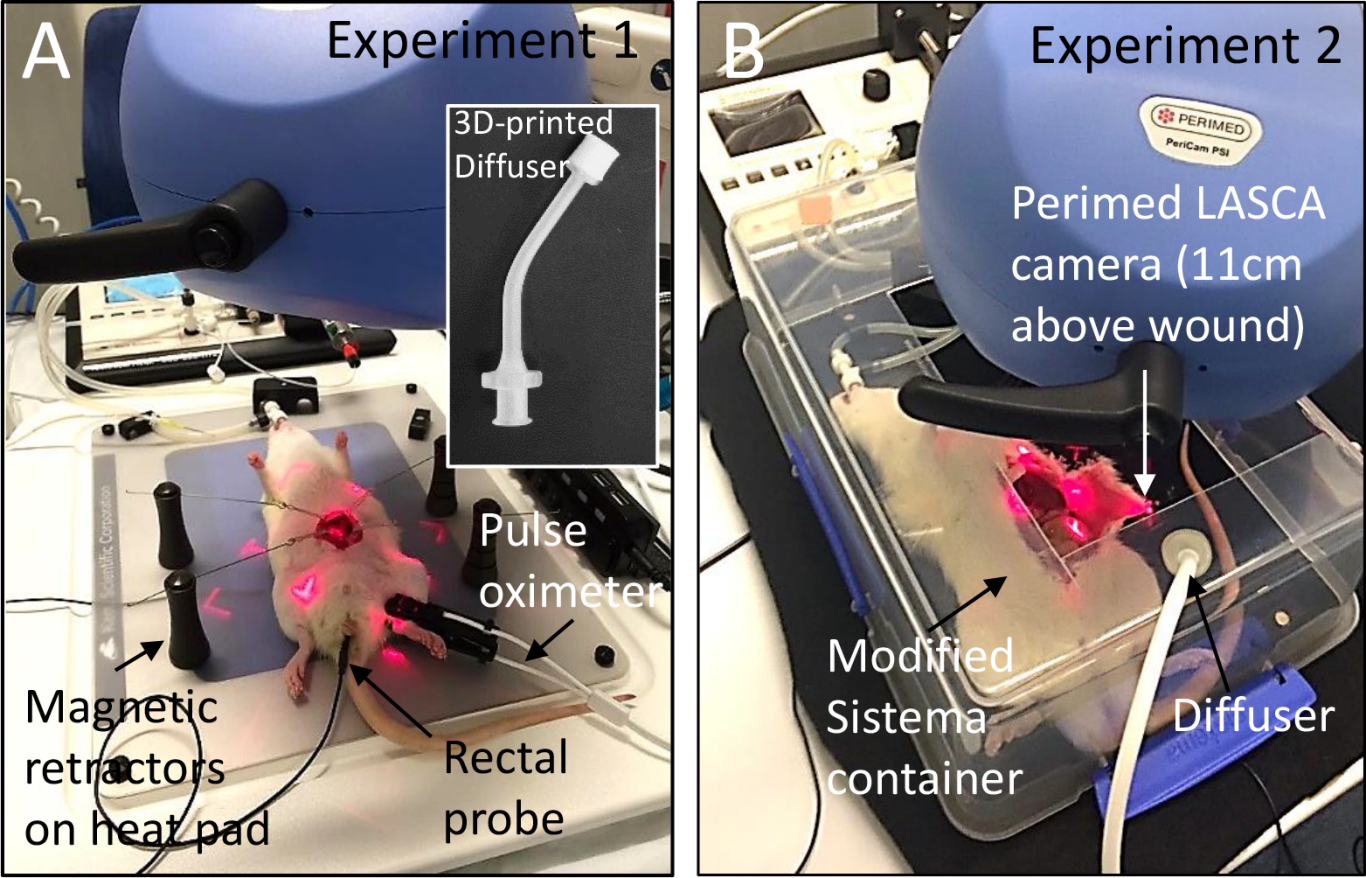
Animal and experimental setup. (A) Experiment 1 setup. Open wound held by magnetic retractors. Inset image illustrates the in-house designed and 3-D printed diffuser used to diffuse CO_2_ into the rat abdominal cavity. Intubated rats were constantly monitored for temperature through a rectal probe and oxygenation through a pulse oximeter and extubation oximeter. Thermal camera images were taken at 11 cm height prior to perfusion camera positioning at t = 0’ and t = 60’. (B) Experiment 2 setup: Intubated rats were covered with the modified Sistema container. The central point of the LASCA laser was positioned adjacent to the viscera, on the abdominal wall flap, for calibration prior to recording. CO_2_ was insufflated into the Sistema container through a pre-inserted VITA-diffuser. The underlying heat pad was not used and a light absorbing, non-reflective black cloth was placed under the rat.

Animals were anaesthetised in an isoflurane induction box (5% isoflurane; 100% oxygen at 1 L/min). Endotracheal intubation was done using a blunt cut down 16G intravenous catheter (Becton Dickinson, New Jersey, USA) with a BioLite intubation system (Braintree scientific, Braintree, MA, USA) and intubation stand (Braintree scientific). Intubated rats were then connected to the SomnoSuite® Small Animal Anaesthesia System (Kent Scientific, Torrington, USA) and anaesthesia was maintained via ventilation with 2.5–3.5% isoflurane in 100% oxygen. Ventilation specifications varied on weight where typically for a 320 gram rat (average weight investigated) there were 72 breaths per minute, 2.4 mL tidal volume, 175 ml/min minute volume and 13 cm H_2_O peak pressure. Depth of anaesthesia was monitored via continuous measurement of heart rate and pulse pressure using the Mouse STAT output, connected to the PhysioSuite system (Kent Scientific) (Fig 1A and 1B). The PhysioSuite system includes the following modules: MouseSTAT—pulse oximetery and heart rate (lower left paw); RightTemp–core body temperature (rectal probe and heating pad set to maintain 37°C); CapnoScan–end tidal CO_2_ (Fig 1A). All procedures were carried out within a bioBUBBLE containment room (BioBUBBLE; Colorado, USA) within the animal facility with ambient room temperatures consistently maintained at 19°C (+/- 1°C) with 55% (+/- 10%) humidity. Intraoperative measurements taken were rectal temperature, SpO_2_, heart rate, MinCO_2_, PeakCO_2_ and respiration rate, recorded every 5 seconds throughout the procedure beginning at time of incision.

### Experimental design and treatment conditions

In this study, we utilised two experimental models to study perfusion and temperature. The first model investigated temperature changes utilizing a miniaturized 3D printed CO_2_ diffuser and magnetic retractors. This allowed for the CO_2_ gas to be insufflated directly into an open abdominal cavity closely mimicking clinical applications (experiment 1). This experiment resulted in unstable perfusion measurements. The underlying heat pad was hypothesized to be a cause for the poor perfusion recordings and so a second model was devised to alleviate this factor. This second model investigated visceral tissue perfusion using LASCA, a non-invasive measurement of perfusion in real time (experiment 2). No retractors were used and the viscera were exposed using a large L-incision to create an abdominal flap. CO_2_ was insufflated into a modified Sistema container covering the entire rodent. Recordings were done on the edge of the viscera where there was reduced abdominal movement from breathing. The underlying heat pad was also turned off to remove any perfusion bias resulting from external heating.

Two experimental conditions were evaluated with the following aims:

- Experiment 1 (three arms; independent interventions): Measure core body temperature using a retracted open abdominal cavity model with the aim of expanding on previously published data showing HH CO_2_ increases core and visceral temperature when used during open abdominal surgery.
- Experiment 2 (repeated measures crossover): Measure abdominal visceral perfusion using a non-retracted abdominal cavity model with the aim of identifying whether HH CO_2_ has an effect on visceral perfusion during open abdominal surgery.

#### Experiment 1

In this experiment we compared the effect of HH CO_2_ (100% CO_2_; 37°C; >98% relative humidity (RH)) to CD CO_2_ (100% CO_2_; 19°C; <0.003% RH) and ambient air (0.04% CO_2_; 19°C; 55% RH) on animal rectal temperature. A total of 15 rats were used in three experimental treatment groups. Group 1: Open abdominal cavity exposed to ambient air (no insufflation; n = 4). Group 2: Open abdominal cavity insufflated with HH CO_2_ (n = 7). Group 3: Open abdominal cavity insufflated with CD CO_2_ (n = 4). Once intubated and ventilated the rats were monitored for core body temperature using a rectal thermometer. During intubation and anaesthetic induction, core body temperature was maintained above 35.5°C with an external heat lamp prior to surgery. Laparotomy was performed with a 4 cm incision along the abdominal midline. The skin and oblique muscles were then retracted using paddled retractors held in place by surgical board magnets (Kent Scientific, Torrington, USA; Fig 1A), creating a wound cavity deep enough to create a local environment of high CO_2_ gas concentration. CO_2_ was continuously insufflated at 300 mL/min through a 3D-printed diffuser that allowed for release of the gas into the abdominal cavity at low velocity and with minimal turbulence (Fig 1A). A CO_2_ flow rate of 300 mL/min was calculated using mass transfer similarity analysis and a surgical humidifier was modified to provide this required flow. The validity of a 300 mL/min flow rate was confirmed by means of smoke visualisation, the methodology of which can be found in [23] and a detailed description of these principles has previously been published [24]. Briefly, the 300 mL/min flow rate was deduced from 0 mm smoke penetration of a 20 mm deep cavity, modelled to approximate the animal cavity. Smoke penetration depth was measured as a normal distance between the top edge of the cavity and the smoke edge using a pixel-calibrated ruler. Humidification was provided through the modified Fisher & Paykel Healthcare surgical humidifier (HumiGard™, Auckland, New Zealand) and gas delivered through an insulated, heated delivery tube. For the CD CO_2_ setup insulation was removed from the chamber and heating wire to better allow the CO_2_ to reach room temperature following release from the CO_2_ bottle. The surgical humidifier was not turned on and flow rates were kept the same. There were no mesentery manipulations. For the purpose of this study CD CO_2_ refers to CO_2_ exiting the bottle with a known RH of <0.003% and equilibrating to room temperature (19°C). For the ambient air experiment both the HumiGard and CO_2_ flow were turned off, leaving the viscera exposed to the ambient environment.

#### Experiment 2

In this experiment we compared HH CO_2_ (100% CO_2_; 37°C; 98% RH) to ambient air (0.04% CO_2_; 19°C; 55% RH) and investigated the effect they had on visceral perfusion. A total of 5 rats were used in this repeated measures crossover experiment, with each animal receiving alternating exposures to ambient air and HH CO_2_. All rats were assigned the same sequence of treatments. Throughout the procedure, animals received alternating exposures of HH CO_2_ (HH CO_2_ turned on) and ambient air (HH CO_2_ turned off). Cold, dry CO_2_ was not investigated in experiment 2. A 5 cm midline incision and two lateral incisions were made to create a tissue flap that would allow for LASCA monitoring of the lateral viscera edge, where torso movement from breathing did not interfere with measurements (Fig 1B). Heat pads were not used in the perfusion experiments to avoid any heat-pad induced perfusion bias during recording.

To model the open cavity a plastic Sistema box was modified by cutting a section out in the base to allow for perfusion monitoring as well as a slit in the side to allow for the intubation tubes to fit. This experimental approach differed to the mini retractor model of experiment 1 so as to avoid the camera “dead spot” of the mini diffuser (the area covered by the diffuser). The modified box was placed over the intubated animal and CO_2_ diffused through a VITA-diffuser™ (Cardia Innovation AB, Stockholm, Sweden) inserted through a small hole at the base of the container, as shown in Fig 1B. The perfusion camera was placed approximately 11 cm above the cavity. For the HH phase CO_2_ was delivered from a D-sized bottle (BOC Ltd, Auckland, New Zealand) and warmed to 37°C and humidified to 98% RH through a Fisher & Paykel Healthcare surgical humidifier (HumiGard™, SH870, Auckland, New Zealand). For ambient air exposure there was no CO_2_ delivery. There were no mesentery manipulations. One hundred percent CO_2_ and >35°C were recorded within the container within 30 seconds of humidifier activation. The Sistema container was lifted slightly during the off phase to evacuate residual CO_2_.

### Perfusion

The PeriCam PSI Laser Speckle Contrast Imager (Perimed AB, Järfälla, Sweden) was used as detailed in [25]. The PeriCam PSI camera measures blood flow through LASCA. Fundamentally, laser speckle detectors count the interference patterns within a tissue generated from diffused light (a speckle). The detector then calculates the spatial and temporal statistics of the speckle pattern using contrast analysis [26]. In relation to blood cells, which are moving through tissues, the speckle pattern is dynamic and thus measurable by Doppler shift (for a detailed description see [25]). The Perimed camera allows for continuous monitoring of dynamic blood flow and records the data as a perfusion unit (PU), a numerical value that increases with increasing perfusion, with a range between 0 and 600.

The LASCA camera was mounted adjacent to the surgical suite and the camera positioned above the abdominal cavity and wound flap. The imaging zone was identified by a red square and a centre dot, positioned in the middle of the cavity (Fig 1B). The perfusion parameters were as follows: sample rate of 1 frame per second and detecting distance of 11±1 cm, which gave a point resolution of 100±10 μm/pixel. Images were captured at 42 images/sec, with averaging every second. Validation experiments were carried out to determine the efficacy of perfusion detection on the abdominal wall tissue flap as well as the exposed viscera. After preliminary analysis, the monitoring area, called the Region of Interest (ROI), was a confined to a 100±10 mm^2^ area of the exposed viscera adjacent to the exposed tissue flap. Camera settings were set to the following: Perfusion scale 10–300 PU; Perfusion filter 40–600 PU; Intensity scale automatic. Pilot measurements of CO_2_ concentration and temperature within the container were made using a CheckMate II gas analyser, (PBI Dansensor, Ringsted, Denmark) and Fluke52 II thermometer (Fluke, USA), respectively. All perfusion data was recorded in real time, and the perfusion units of the ROI were calculated and analyzed using PIMSoft version 1.5 software (Perimed AB, Järfälla, Sweden).

### Temperature

Core body temperature was recorded via a rectal thermometer placed >2 cm into the rectum, with measurements recorded every 5 seconds. Rectal temperatures are reflective of core colonic temperatures when probes are placed at this depth in the rat [27]. Abdominal cavity visceral temperature was measured via thermography using a FLIRC2 compact thermal imaging camera (FLIR systems, Boston, MA, USA). Thermal images were taken at time t = 0’ and t = 60’. Visual assessment of tissue drying/moistening was done using images from an 8 megapixel camera (Apple, USA).

### Statistics and analysis

Animal profiles were analysed in one-way ANOVA. For core body temperature the data were analysed as follows: Subsequent temperature readings for each experiment were differenced and summed and then the values analysed by a student’s t-test with Welch’s correction. The Shapiro-Wilk test was used to test the normality of the data. Central tendency measures used were means and standard error of the mean (SEM). P-values of <0.05 were considered statistically significant. Graphical processing was done using GraphPad Prism (GraphPad Software, La Jolla, USA).

For perfusion analyses data was analysed as follows: A total of 5 rats were used in a repeated measures crossover trial, with each animal receiving alternating exposure to ambient air (CO_2_ turned off) and HH CO_2_ (CO_2_ turned on) at least five times over a period of at least 90 minutes. To determine the average linear effect of the 2 treatments on blood flow over the region of interest, a nested mixed effects (repeated measures) model was fitted to the time series data for each rat:

$${ROI}_{ij}=X_{ij}\beta +Z_{ij}b_{ij}+\epsilon_{ij}$$

$$b_{ij}∼Ɲ\;(0,D)$$

$$\epsilon_{ij}∼Ɲ\;(0,\Sigma )$$

ROI_*ij*_ is the vector containing the time series of blood flow measurements over the region of interest for the i^th^ rat and the j^th^ treatment alternation. X_*ij*_ is the matrix containing the covariates for estimating the fixed-effects, which includes time, treatment, and their interaction (and a column of 1’s for the intercept). The parameters of the fixed effects are contained within the β column vector. Z_*ij*_ and b_*ij*_ are the design matrix for the random effects, and their parameters respectively. The model allows for a random intercept and random slope (for the ROI vs. time relationship) for each treatment alternation nested within each rat. The b_*ij*_ and ϵ_*ij*’_s are normally distributed with variance-covariance matrices D and Σ respectively. An advantage of this model formulation is that the induced correlation structure (in D) accounts for the temporal autocorrelation. All analyses were conducted using the R language and environment for statistical computing ([28], v3.4.3). The *nlme* R package was used to fit the mixed effects model, and the *tidyverse* suite of R packages were used for all the data manipulation and preparation.

## Results

### Animal profiles

In experiment 1 (n = 15) there were no significant differences in age (p = 0.452) or weight (p = 0.504) within the treatment groups. Rats used in the repeated measures crossover perfusion experiment 2 (n = 5) were similarly equivalent in age (58.6 ± 2.88 days) and weight (338.6 ± 3.51 g).

### Experiment 1: Core body temperature using a surgical open abdominal cavity model

Validation of the 3D-printed miniaturized gas diffuser was carried out using smoke penetration of an artificial cavity modelled to represent the rodent open abdominal cavity. Different flow rates were tested: 30 mL/min flow gave a smoke penetration of 8.2 mm, 100 mL/min gave a penetration of 4.7 mm, and 300 mL/min gave a penetration of 0 mm (S1A–S1C Fig). Based on these validation results a 300 mL/min CO_2_ flow rate used throughout experiment 1.

**Fig 2:**
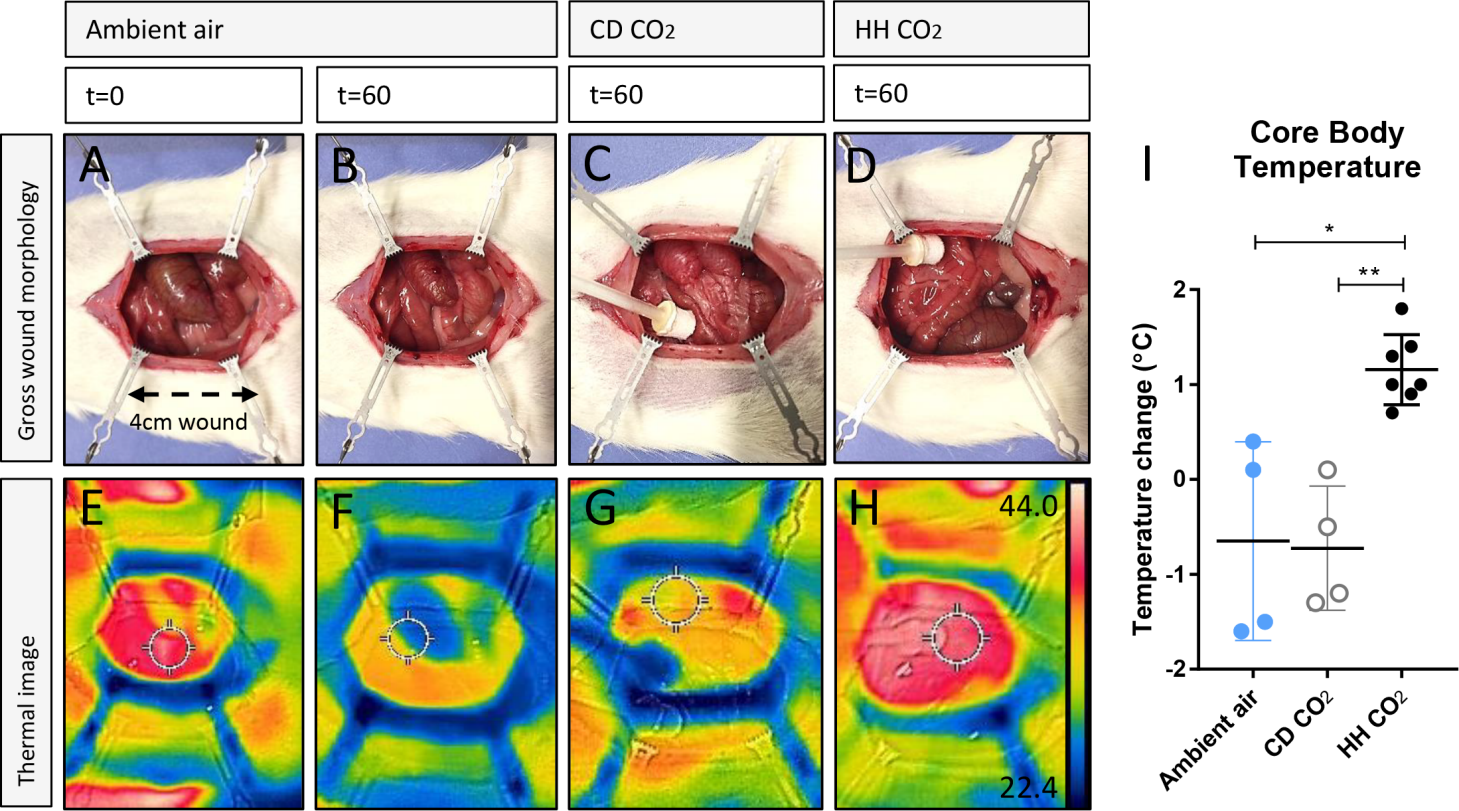
Insufflation with heated, humidified CO_2_ into an open abdominal cavity increases visceral and core body temperature. (A-D) Camera images of magnetic retracted open abdominal cavities created in rodents receiving standard treatment (ambient air), cold, dry (CD) CO_2_ or heated, humidified (HH) CO_2_. Images were taken at the start of surgery after the abdomen was initially opened (t = 0’) and at 60 minutes (t = 60’). Abdominal cavities were 4 cm in longitudinal length. The 3D-printed diffuser was inserted in the cranial edge of the wound. (E-H) Thermal images of the open abdominal cavity at times correlating to the images shown. Values in H are degrees centigrade correlating to the thermal scale shown adjacent. (I) Average cumulative core body temperature change over 60 minutes. Horizontal lines indicate where statistical differences were identified; * indicates p<0.05 for HH vs.CD; ** p<0.01 for ambient air vs. HH.

Upon initially opening the abdominal cavity the visceral and parietal peritoneum appeared moist and warm as shown by photographic and thermal imaging (Time t = 0’) (Fig 2A and 2E). Following 60 minutes exposure to ambient air the peritoneum and organs appeared very dry and cool (Fig 2B and 2F). This drying was exacerbated in rats receiving CD CO_2_ (Fig 2C and 2G) but ameliorated in rats receiving HH CO_2_ (Fig 2D and 2H). Still photographs of the thermal images identified that abdominal cavity visceral temperatures were maintained (no heat loss) in the HH CO_2_ group (Fig 2E and 2H). In comparison, viscera temperatures appeared cooler in the CD CO_2_ group (Fig 2G), and an even cooler visceral temperature was observed in the ambient air group after 60 minutes (Fig 2F).

Core body temperature was significantly increased in rats receiving HH insufflation compared with ambient air, with a rectal temperature disparity of 1.8°C, HH +1.15 ± 0.14°C vs. ambient air -0.65 ± 0.52°C; p = 0.037, (Fig 2I). Compared with CD CO_2_ insufflation -0.73 ± 0.33°C, rats receiving HH CO_2_ were significantly warmer (p = 0.0057; Fig 2I).

Perfusion measurements of the exposed viscera were conducted with scores ranging between 250–400 PU across all rodents. The high PU’s recorded within the cavity resulted in an inability to detect any perfusion changes using this model. Specific causes for the high PU recordings are unknown but hypothesised to be associated with the continuous external heating from the underlying heat pad, which was on throughout the experiment.

### Experiment 2: Visceral perfusion using a non-retracted open abdominal cavity model

**Fig 3:**
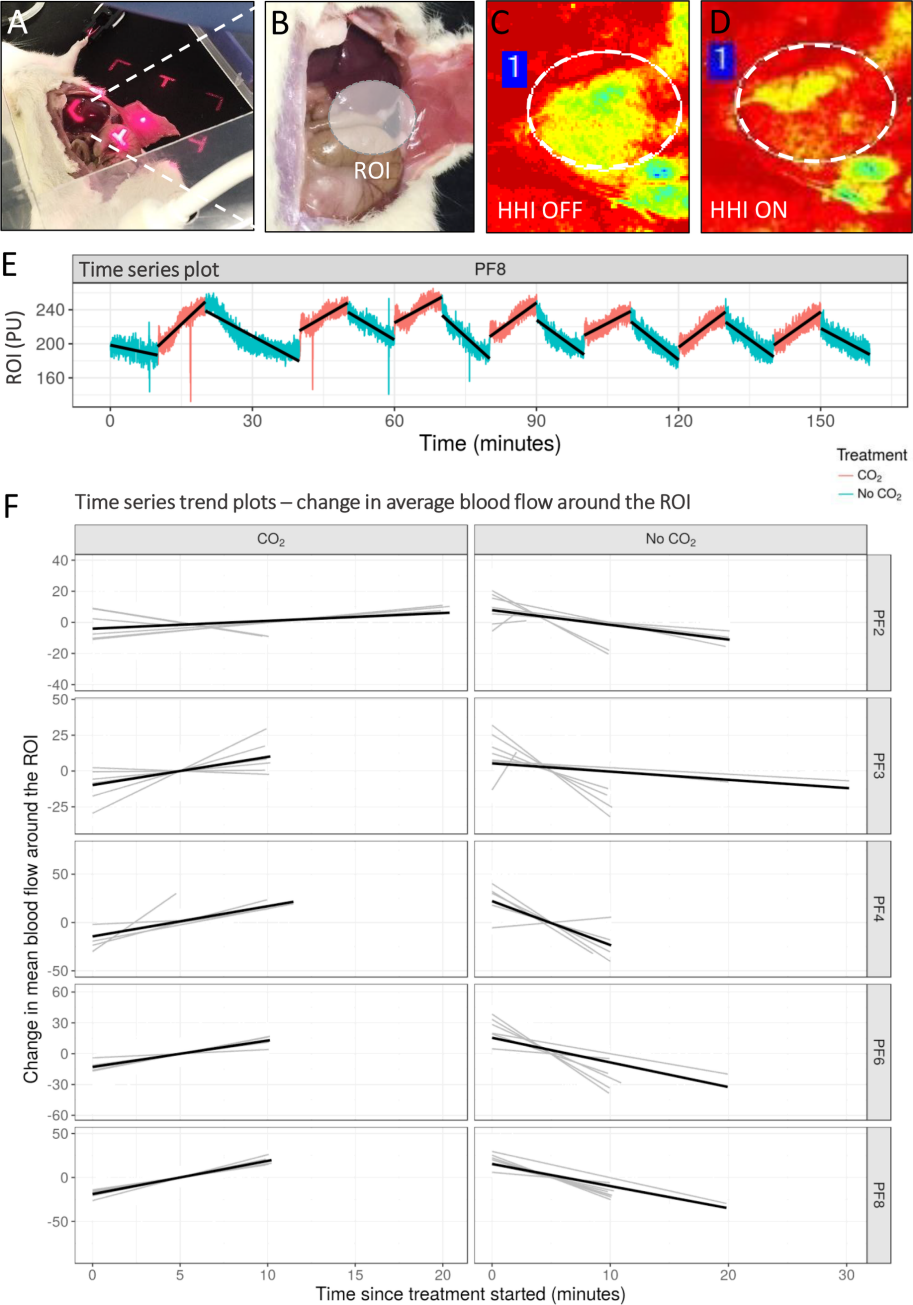
Insufflation with heated, humidified CO_2_ increases visceral tissue perfusion. (A, B) Perfusion was recorded 11 cm above the abdominal cavity. The ROI was a 100 mm^2^ circular section of the viscera, adjacent to the extended flap (inset). (C, D). Analysis of perfusion was recorded via a colorimetric perfusion unit scale. Measured ROI shown as a dotted circle. HHI, heated, humidified insufflation. (E) Time series plot showing the response of the ROI to CO_2_ (red) and no CO_2_ (blue) for one of the tested rodents. Black lines represent the trend line. (F) Time series plots showing the change in the average amount of blood flow around the ROI (light grey lines), and the associated trend lines for each time period and each treatment group, for each rat (dark grey linear trend lines). A trend line for each rat and treatment group combination (black linear trend line) is also indicated to visualise the overall trend in each of these facets. PF2, 3, 4, 6, and 8 are the tested rodents identification.

Preliminary results of perfusion measurements of the exposed abdominal wall flap were very low. In contrast to the over-exposed viscera in experiment 1, the abdominal flap perfusion measurements were too low to detect changes in perfusion over time. To this end, perfusion of a 100±10 mm^2^ area of the lateral viscera (ROI) was consistently monitored for each animal (Fig 3A–3D).

Time series plots identified clear differences in perfusion trends when CO_2_ was turned on and off (Fig 3E and 3F light grey lines). Comparisons of the linear slopes across all rodents identified strong evidence of a real treatment effect (interaction p<0.0001; Fig 3F black lines). When HH CO_2_ was turned on the blood flow over the ROI significantly increased by 2.4 units/min (95% CI 1.23–3.58; p<0.0001) (Fig 3E and 3F). When HH CO_2_ was turned off the blood flow over the ROI significantly decreased by 5.2 units/min (95% CI -6.83–3.58; p<0.0001) (Fig 3E and 3F).

To avoid confounding exogenous heating effects on the perfusion measurements the heating pads and associated temperature monitoring were not used for this experiment.

## Discussion

Cold, dry CO_2_ insufflation is currently the standard method of insufflating the pneumoperitoneum during laparoscopic surgery but many studies have revealed underlying damage is incurred when using CO_2_ straight from a bottle [29–31]. To oppose the damaging effect of CD CO_2_ on the mesothelial cells that line the peritoneum, many clinicians have turned to heating (37°C) and humidifying (>90% RH) the CO_2_ during insufflation. HH CO_2_ has been shown to significantly increase intraoperative core body temperature [32], reduce post-operative pain [33] and reduce analgesic requirement [34]. Subsequent human trials have shown that heating and humidifying CO_2_ can significantly ameliorate the damages induced by CD CO_2_ as well as prevent hypothermia frequently seen in laparoscopic procedures [35–40]. Similarly, the use of HH CO_2_ during open surgery has been shown to significantly increase surgical wound and core body temperature [21, 22].

We showed in our open abdominal rodent study that HH CO_2_ insufflation significantly increased core body temperature compared with ambient air and CD CO_2_. This finding is consistent with previous studies, where CD CO_2_ insufflation decreased core body temp during open abdominal surgery [41]. Furthermore, thermal imaging indicated that visceral temperature appeared higher in open cavities insufflated with HH CO_2_ compared with ambient air or CD CO_2_, results similarly observed in human cases [21, 22]. This study also reported that insufflation of HH CO_2_ ameliorated subjective observations of desiccation of the abdominal tissue surface. Our results are in line with other recently published data that show that exposure of the open abdominal cavity to ambient air causes microscopic damage to the peritoneal mesothelium, and that this is prevented with insufflation of HH CO_2_ [42]. These results are also consistent with animal studies of laparoscopy, that showed drying and desiccation of the peritoneum, as well as a significantly increased rate and incidence of malignant tumor implantation following brief exposure to CD CO_2_ [30, 31, 43, 44].

To our knowledge, this is the first research to investigate the effect of abdominal HH CO_2_ insufflation on visceral perfusion during open abdominal surgery, and provides an explanation for the increase in PtO_2_. Furthermore, we maintained core body temperature as previously published [16, 45]. HH and CD CO_2_ have been shown to increase PtO_2_, however the specific mechanism of how CO_2_ gas increases PtO_2_ was not explored. Marshall et al. (2015) showed that compared with ambient air, HH CO_2_ approximately doubled tissue oxygen tension, while CD CO_2_ insufflation increased PtO_2_ by only 50% [45]. This may suggest that modifiers of the gas, temperature and humidity, are as important in increasing PtO_2_ as the CO_2_ itself. This could be explained, at least in part, by the Bohr Effect. As CO_2_ is insufflated into the abdominal cavity the partial pressure of CO_2_ in the blood increases, which leads to a decrease in blood pH resulting in a lower affinity of haemoglobin to oxygen [46]. The right-ward shift in the oxygen-haemoglobin dissociation curve yields an unloading of oxygen, which increases PtO_2_. This effect is further enhanced by increased temperature, which shifts the oxygen-haemoglobin dissociation curve even further to the right. While CD CO_2_ wasn’t investigated in this perfusion study we believe that differences induced by the temperature of the CO_2_ would not be detected in this experimental setup due to the potent vasodilatory effect of CO_2_, which would overshadow any temperature changes that could be detected with the LASCA camera used in this study. Preliminary perfusion investigations carried out in this study had already identified temperature-sensitivity issues of the camera. While temperature and humidity will likely act to increase perfusion further, more studies with equipment allowing for higher LASCA resolutions are required to confirm this. In a recent publication authors successfully modified post-processed, binary perfusion data collected from a high resolution laser speckle contrast imaging (LSCI) camera. In that report, using an enhancing algorithm Gnyawali et al. (2017) were able to monitor perfusion in the vascular network of the murine dorsal skin, circumventing the sensitivity issue observed using the LSCI camera alone [47]. This algorithm may be useful to get around the sensitivity issues we observed, enabling a more widespread perfusion analysis in future studies.

The difference in visceral perfusion during surgery, resulting from CO_2_ conditioning, may have important implications for clinical practice. Poor tissue perfusion has been identified in patients with hypertension, obesity and diabetes mellitus [48], which are all correlated with lower PtO_2_ and worsened outcomes following surgery. Reduced PtO_2_ recorded in obese patients has been shown to be associated with SSI onset [49]. Treatment options available to deal with falling tissue pressure and oxygenation are largely vasopressors and inotropes, however their uses have many unwanted caveats including deleterious thermogenic effects and reduced cellular energy [50, 51]. Furthermore, such treatment options can promote bacterial growth [52, 53] and accelerate biofilm generation which increases the risk of SSI [54]. One study found a significant increase in the risk of SSI when intraoperative vasopressors were administered during liver transplantation [55], and evidence has been published indicating that fewer bacteria are required to initiate a SSI where tissue perfusion is low [56, 57]. Increasing local tissue perfusion through insufflation with HH CO_2_ offers a method of increasing local and core body temperature, and possibly also decreasing SSI rates. Previous research showed that HH CO_2_ insufflation in a rat model increased PtO_2_ by 30 mmHg compared with exposure to ambient air [45]. This is significant as a 25 mmHg increase in PtO_2_ has been reported to predict a 30% drop in the rate of SSI [9]. The current research suggests the increase in tissue oxygenation is at least partially achieved by an increase in tissue perfusion.

In addition to its immunogenic effects, proper perfusion is an essential requirement for reliable healing of gastrointestinal anastomoses. One of the more difficult anastomoses of all gastrointestinal operations is the cervical esophagogastric anastomosis done during certain gastric reconstructions. A critical relationship between good perfusion and anastomotic healing has been shown in esophagectomy using laser-assisted Indocyanine green fluorescent dye angiography (LAA) [58]. This approach was used to counter the weakness of micro perfusion monitoring by the more typically used Doppler signalling for gastric perfusion measurements. However, LAA is largely qualitative and lacks quantitative reliability [58]. Furthermore, LAA and laser Doppler techniques do not allow for simultaneous assessment of adequate blood flow in regions of interest, a technique possible with LASCA. Recently, one studied successfully showed that LASCA imaging could identified ischemic areas on gastric tube anastomoses following esophagectomy, establishing its reliability in measuring perfusion during anastomotic healing [59].

Here we used a LASCA camera to accurately record, through a colorimetric assay, the change in perfusion in an open abdominal cavity. This approach to measuring tissue perfusion has been successfully applied to monitor blood flow in the hand [26], eye [60], and the neocortex [61]. A number of studies have used LASCA to study perfusion in rodents [62–65], however, its use in human studies is only just emerging [59, 62, 66]. We initially attempted to record visceral perfusion in the retracted wound model (experiment 1) but we experienced sensitivity issues with the camera. In this experiment the perfusion measurements were too high, with too much background noise to detect any changes induced by insufflation. In addition, we found that the sensitivity of the LASCA perfusion camera to detect perfusion shifts in thin tissues was difficult. Our attempts to detect perfusion changes in the parietal tissue flap of a rat were challenged by these sensitivity issues, and while perfusion changes in the flap showed preliminary results similar to the viscera recordings, the location of these changes within the flap were inconsistent. This is likely due in part to the sensitivity of the laser within the Perimed camera, which has a minimum resolution of 0.1 mm/pixel; a resolution too small to accurately detect perfusion changes in a ~3 mm thick tissue flap. To this end we chose to investigate a 100±10 mm^2^ area of viscera so as to confine our ROI to a more vascularized location, where CO_2_ insufflation would allow for the recording of consistent perfusion data over time in all rodents.

Using a repeated measures analysis, we were able to show that blood flow significantly increased within the ROI when HH CO_2_ was insufflated into the abdominal cavity. Furthermore, blood flow significantly decreased within the ROI when CO_2_ insufflation was removed. There are, however, some cautionary limitations of using a repeated measures model. The estimate of the intercept in the analysis could not be used as an estimate the average blood flow over the ROI in rats, because this estimate was a result of the arbitrary alternation of the treatment effect throughout the experiments. Nevertheless, this had no effect on the estimate of the treatment effects through time. In addition, the estimates given in this study are only an estimate of the linear effect of the treatment. While the blood flow through the viscera will have a maximum and minimum value that would differ among animals, the experiment was not conducted to allow for a curve of best fit analysis to more-reliably estimate the treatment effect with these upper and lower bounds. This means that the linear estimate is only useful to describe the average treatment effect when the blood flow is at or near its maximum or minimum. Nevertheless, the statistical model used here confirmed the hypothesis that HH CO_2_ significantly effects the change in perfusion over time.

In addition to the known sensitivity issues, the insufficient knowledge and published experience of the use of LASCA to measure perfusion during surgery currently limits its widespread application. Compared to conventional laser Doppler imaging, which has excellent spatial resolution, LASCA provides a means to better visualise rapid perfusion changes over time at the expense of contiguous resolution. LASCA achieves its better temporal resolution because the calculation of the speckle is done using a full-field technique, while laser Doppler imaging uses a scanning technique which has longer measurement times. While newer Doppler imagers have the means to carry out a full-field technique using a high speed CMOS camera, these are expensive and the advent of newer LASCA imagers, such as the one used in this study, are further increasing the speed of speckle contrast calculations [25]. As shown in this investigation, LASCA perfusion measurements provide a fast, sensitive means to investigate perfusion changes in real time, but further studies are required before the technology can be more widely adopted, especially in thin tissues where measurements can be difficult to obtain.

In conclusion, this study showed that insufflation of an open abdominal cavity with HH CO_2_ significantly improved visceral tissue perfusion compared with exposure to ambient air. These results help to explain previously published literature by proposing a mechanism by which HH insufflation gas increases blood delivery to the viscera, and furthers the understanding of a simple therapy that may reduce the rates of surgical site infection and improve post-operative recovery.

## Supporting information

S1 Fig Three hundred mL/min provides complete insufflation of a rodent size abdominal cavity model. (A-C) Smoke visualization experiments illustrating smoke penetration of a 20 mm deep rodent abdominal cavity model with 30 mL/min, 100 mL/min and 300 mL/min CO_2_ insufflation. Table indicates recorded smoke penetration depth as a function of insufflation flow rates.
(TIF)
