# Supplementary figures and images for: Increased visceral tissue perfusion with heated, humidified carbon dioxide insufflation during open abdominal surgery in a rodent model
Source: PLoS One. 2018 Apr 4;13(4):e0195465. doi: 10.1371/journal.pone.0195465 (PMC5884566; doi:10.1371/journal.pone.0195465)

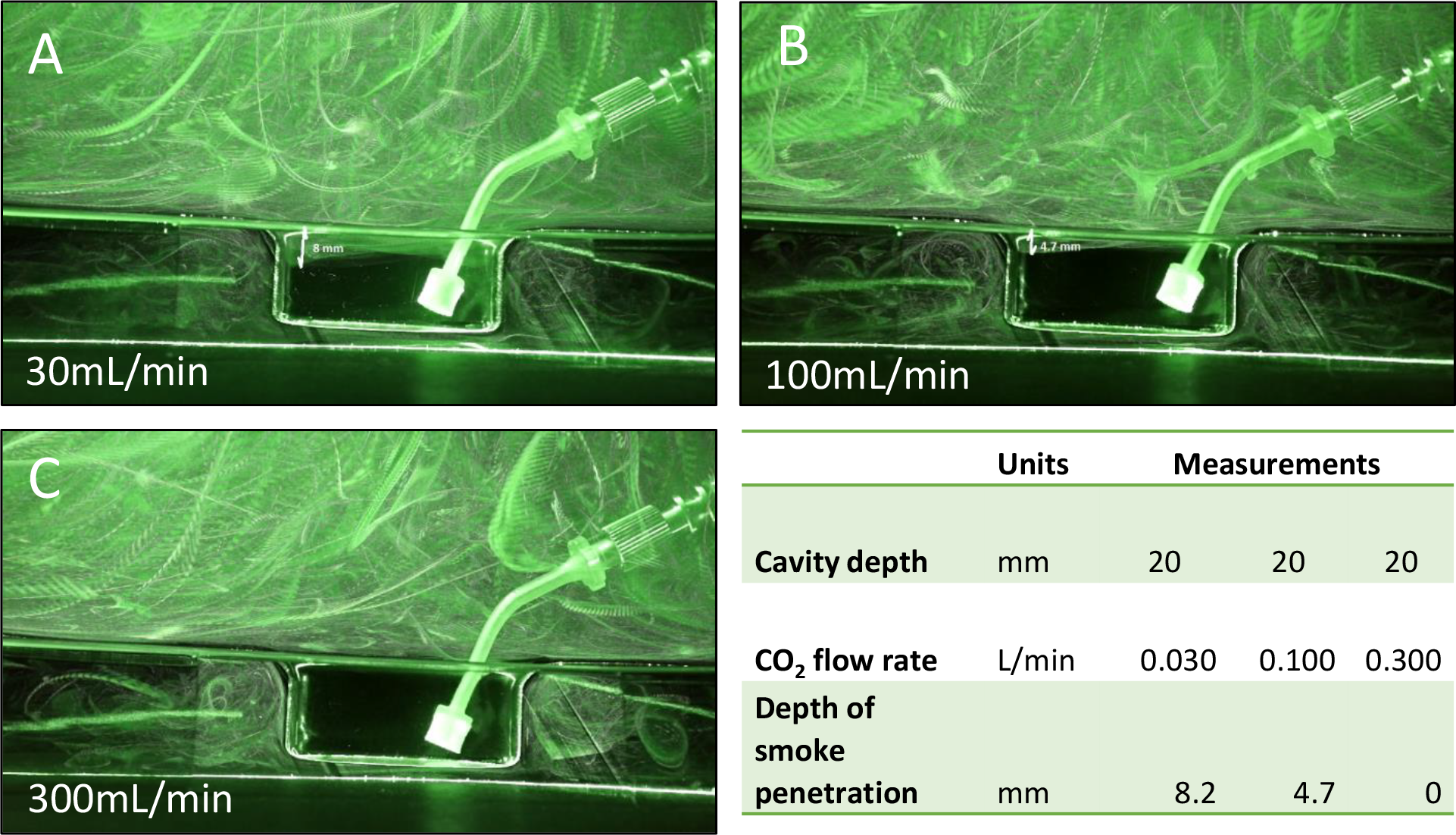

Supplement: S1 Fig — (A-C) Smoke visualization experiments illustrating smoke penetration of a 20 mm deep rodent abdominal cavity model with 30 mL/min, 100 mL/min and 300 mL/min CO2 insufflation. Table indicates recorded smoke penetration depth as a function of insufflation flow rates. (TIF) [file pone.0195465.s001.tif]
